# Case report: Solitary mass of the sciatic nerve confirmed as a primary extranodal manifestation of diffuse large B-cell lymphoma in a geriatric patient

**DOI:** 10.3389/fonc.2024.1354073

**Published:** 2024-03-22

**Authors:** Hannes Becker, Antonio Vogelsberg, Daniel Feucht, Arne Estler, Deniz Tafrali, Jens Schittenhelm, Jakob Milla, Sylvia Kurz, Falko Fend, Marcos Tatagiba, Martin U. Schuhmann, Helene Hurth

**Affiliations:** ^1^ Department of Neurosurgery, University Hospital Tuebingen, Eberhard Karls University Tuebingen, Tuebingen, Germany; ^2^ Department of Neurology & Interdisciplinary Neuro-Oncology, Hertie Institute for Clinical Brain Research, Center for Neuro-Oncology, Comprehensive Cancer Center, University Hospital Tuebingen, Eberhard Karls University Tuebingen, Tuebingen, Germany; ^3^ Department of Pathology and Neuropathology, University Hospital and Comprehensive Cancer Center Tuebingen, Eberhard Karls University Tuebingen, Tuebingen, Germany; ^4^ Diagnostic and Interventional Neuroradiology, Department of Radiology, University Hospital of Tuebingen, Eberhard Karls University Tuebingen, Tuebingen, Germany; ^5^ Department of Neuropathology, University Hospital Tuebingen, Eberhard Karls University Tübingen, Tuebingen, Germany; ^6^ Division of Pediatric Neurosurgery, Department of Neurosurgery, University Hospital of Tuebingen, Eberhard Karls University of Tuebingen, Tuebingen, Germany

**Keywords:** B-cell lymphoma, sciatic nerve, peripheral nerves, peripheral non nerve sheath tumor, extranodal manifestation

## Abstract

**Background:**

Neoplastic lesions affecting peripheral nerves are rare in the general population and, most often, are benign peripheral nerve sheath tumors. However, a minority of lesions represent high-grade malignancies associated with a poor prognosis, such as malignant peripheral nerve sheath tumors (MPNSTs). Very rarely, these tumors represent peripheral non-nerve sheath tumors (PNNSTs), such as hematological neoplasms that impair nerve function. These can be hard to distinguish from MPNSTs and other lesions arising from the nerve itself. In the present case report, we describe a rare case of direct infiltration of nerves by tumor cells of a hematological neoplasm.

**Methods:**

We report the case of a 90-year-old woman with acute onset of right-sided foot palsy, sensory loss, and pain, caused by an extensive solitary mass of the sciatic nerve in the thigh. We present and discuss the clinical presentation, multimodal diagnostic procedures, and treatment.

**Results:**

MRI of the right thigh and the caudal pelvis revealed a contrast-enhancing lesion infiltrating the sciatic nerve. Additionally performed staging imaging was non-revealing. After multidisciplinary discussion in the neuro-oncology tumor board, a MPNST was suspected and the patient underwent radical tumor resection. However, final histopathology revealed a diffuse large B-cell lymphoma (DLBCL). The patient received adjuvant palliative local radiotherapy which led to acceptable symptom control.

**Conclusion:**

Rare PNNSTs, including extranodal manifestations of DLBCL can have similar clinical and radiological diagnostical features as PNSTs. Comprehensive diagnostic workup of contrast-enhancing lesions affecting peripheral nerves including MRI and metabolic imaging are recommended. Discussion in interdisciplinary tumor boards facilitates finding individual treatment approaches.

## 1 Introduction

Neoplasms affecting peripheral nerves can be divided into tumors arising from the nerve itself (peripheral nerve sheath tumors [PNSTs]) and tumors that arise from tissues other than the nerve sheath but nevertheless impair nerve function (peripheral non nerve sheath tumor [PNNSTs]).

While PNSTs represent approximately 12% of all benign soft tissue tumors, isolated PNNSTs are very rare (1, 2). The majority of PNSTs are benign and histologically represent schwannomas or neurofibromas (1, 3). Diagnostic work up includes ultrasound assessment, if accessible, followed by MRI imaging (4, 5). Surgical removal aiming for preservation of nerve function is the therapy of choice in symptomatic and benign lesions (4). However, a minority of peripheral nerve lesions are either malignant or at a precursor state to malignancy, for example atypical neurofibromatous neoplasms with uncertain biologic potential. It can be radiologically challenging to identify these (4–6). Malignant PNSTs (MPNSTs) are linked to cancer predisposition syndromes like neurofibromatosis type 1 (NF1) (7).They account for 5-10% of all soft tissue sarcomas and are associated with an unfavorable prognosis despite radical surgical resection and multimodal therapy regimens (1, 8, 9).

In contrast, PNNSTs comprise of lipomas, vascular tumors, as well as uncommon presentations of hematological neoplasms such as extranodal manifestations of lymphomas, historically referred to as neurolymphomatosis (10). The latter has been described to exhibit similar radiological features as MPNSTs or PNSTs.

In the following report, we present a rare extranodal manifestation of a diffuse large B-cell lymphoma (DLBCL) manifesting as a PNNST affecting the sciatic nerve function (10).

## 2 Case presentation

A 90-year-old female presented with new-onset weakness and sensory loss of her right lower extremity. Initial neurological examination revealed right-sided plegia of foot extension and flexion with moderate leg pain as well as loss of the superficial sensory function including hypo- to anesthesia and reduced proprioception in the lateral aspects of the calve up to the dorsum of the right foot, consistent with a complete loss of function of the sciatic nerve. The symptoms had developed over two to three days. There were no other neurological symptoms or signs of neurological dysfunction. Clinical examination revealed no lymphadenopathy or splenomegaly. No signs of fever or increased weight were observed over the last months. The patient’s past medical history included hypertension, mild cognitive impairment in the context of confirmed Alzheimer’s disease, and diabetes type 2, yet the patient was still able to live independently. The medical history of the patient’s family was negative for hematological disorders or other tumors.

**Figure 1:**
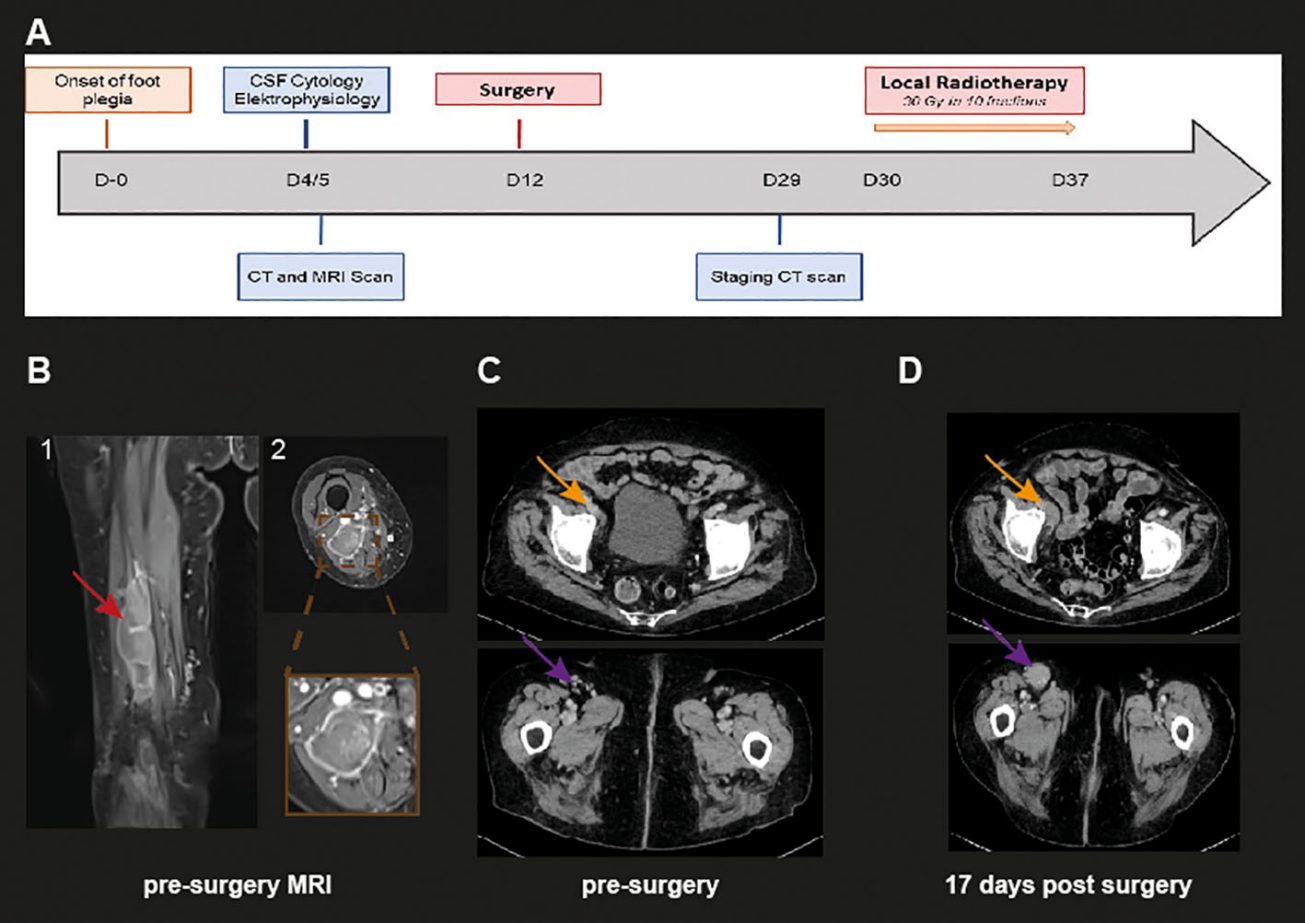
Clinical history and representative radiological images. **(A)** Clinical course and diagnostic work-up of the described case. Day 0 is defined as the day when paralysis of extension of the right foot was evident for the patient. **(B)** Representative coronal (1) and axial (2) MRI images of the right tigh, showing an inhomogeneous contrast-enhancing lesion (red arrows). The brown box depicts a close-up image of the axial tumor expansion **(C)** Representative axial and coronal CT image of the initial CT scan on day 4 revealing no further nodular DLBCL lesions. **(D)** Representative axial and coronal CT image 17 days post-surgery showing novel malignant lesions in the ventral right femoral compartment (top orange arrow) as well as in the right inguinal region (bottom violet arrow).

Initially, the primary concern was that of an ischemic or hemorrhagic vascular event, however, a cerebral CT and MRI did not show evidence for an acute neurovascular event. Given that the presented neurological syndrome was consistent with a peripheral nerve injury, further workup included an electromyogram and nerve conduction studies as well as peripheral nerve ultrasound. The electromyogram showed pathological spontaneous activity of the M. tibialis anterior and the M. gastrocnemius, consistent with an axonal injury to the right sciatic nerve (**Figure 1A**). Peripheral nerve ultrasound revealed a large mass at the proximal to middle part of the sciatic nerve with inhomogeneous echogenicity and signs of a central hematoma. Femoral CT and MRI scans confirmed an inhomogeneous contrast-enhancing lesion of 20 cm length and 2 to 3 cm in diameter originating from the sciatic nerve in the thigh and extending downwards to the popliteal fossa with possible attachment to the femoral veins. Discrete perifocal edema was also observed. In total, imaging was suggestive of a malignant process and compatible with a MPNST (**Figure 1B**). The initial CT scan did not show additional contrast-enhancing lesions in particular in the caudal pelvic region and no enlarged lymph nodes (**Figure 1C**). A lumbar puncture was unremarkable with no evidence of malignant cells (**Figure 1A**). Blood tests showed no evidence of leukocytosis or acute inflammation.

The case was discussed at our interdisciplinary neuro-oncology tumor board. Given the patient’s age but good general condition and the suspected malignancy of the lesion, the options of either radical surgical resection followed by adjuvant therapy, which could provide good local tumor control and possible pain control, or a less invasive approach of palliative local radiotherapy were considered. Following a detailed discussion about the advantages and disadvantages of both approaches, the patient opted to proceed with radical surgical resection. Considering the patient’s advanced age with expected slow axonal recovery and the size of the lesion and the already extensive destruction of the sciatic nerve, nerve reconstruction using sural nerve grafts was not offered.

Surgery was performed in prone position. The tumoral mass was exposed over 30-40 cm and was carefully dissected aided by intraoperative nerve stimulation to preserve the proximal unaffected muscle branches of the hamstrings. The tumor was of hard consistency and infiltrated several smaller nerve branches, including the tibial and peroneal nerves even after the branching of the sciatic nerve and beyond the expected tumor extension based on MRI. Next, the tumor was completely dissected from the femoral veins and the popliteal artery. The sciatic, the tibial and the peroneal nerves were cut in an area considered to be free of tumor and the whole tumor mass was removed en bloc. Following resection, no macroscopical tumor residuals could be identified. Postoperatively, no new sensorimotor deficits were discovered. Pain was initially controlled with peridural anesthesia and later sufficiently treated with oral pain medication. The patient could be continuously mobilized and was mobile on a walker with a food orthesis at the time of discharge from the hospital.

**Figure 2:**
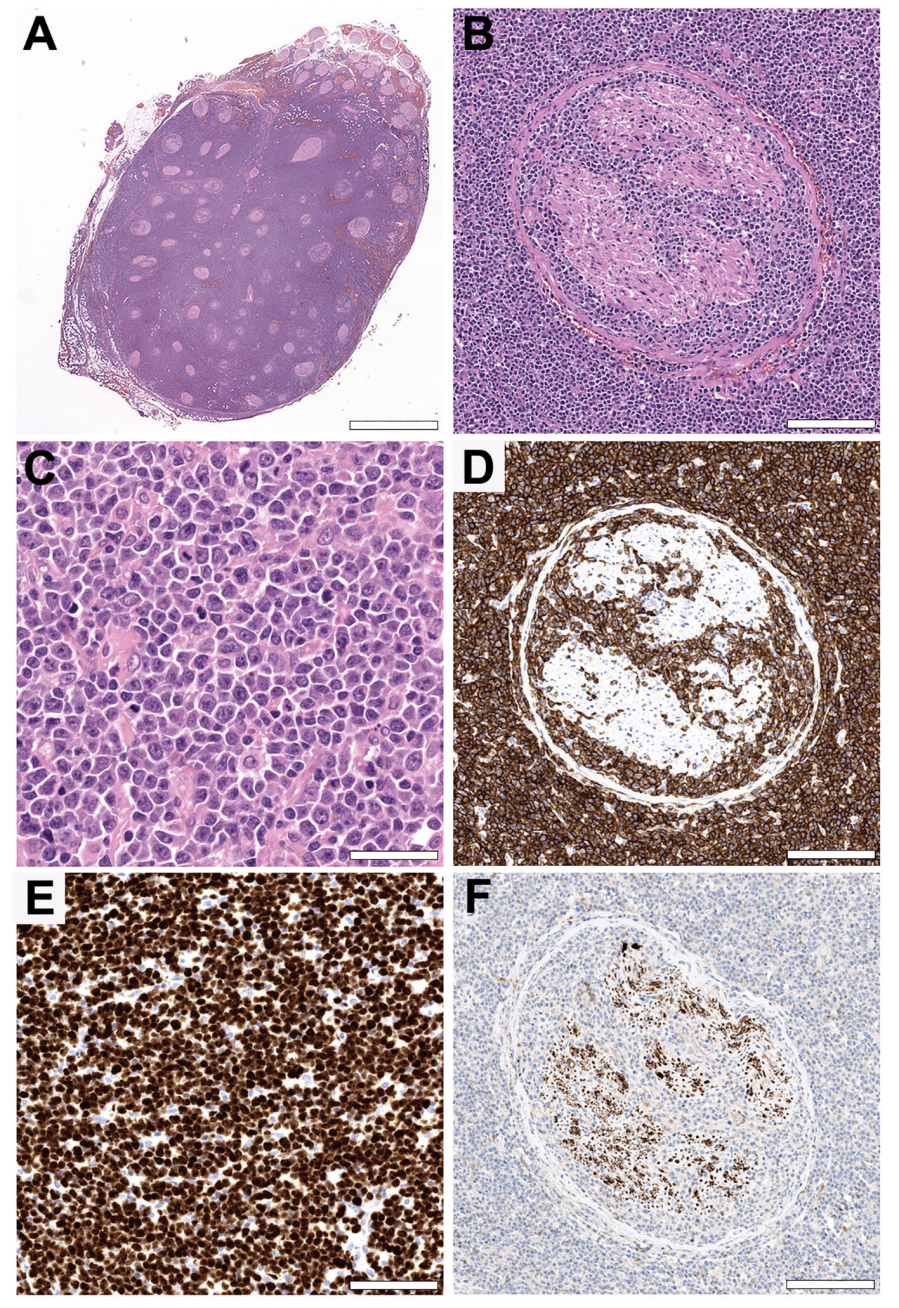
Histopathology. **(A, B)** Hematoxylin and eosin (H&E) stained cross section of the sciatic nerve showing a highly cellular neoplasm growing diffusely in between and within the nerve fascicles. (Original magnifications x5 and x100. Scale bars = 3,75 mm and 170 µm). **(C)** The tumor is composed of medium- to large-sized lymphoid cells with open nuclear chromatin, a single to multiple prominent eccentric nucleoli, and variable amounts of basophilic cytoplasm. Note the high mitotic activity. (Original magnification x400. Scale bar = 45 µm). **(D)** The tumor cells show strong CD20 expression by immunohistochemistry, consistent with a B-cell origin. (Original magnification x100. Scale bar = 170 µm). **(E)** MIB1 staining showing a proliferation rate of more than 90%, in line with the abundance of mitotic figures in the H&E stain. The non-proliferating cells in between are mostly endothelial cells and fibroblasts. (Original magnification x200. Scale bar = 75 µm). **(F)** A neuron specific enolase stain highlighting the reduced number of remaining nerve fibers. (Original magnification x100. Scale bar = 170 µm).

Histologically, the resected sciatic nerve was diffusely infiltrated by a highly cellular neoplasm composed of medium- to large-sized lymphoid cells with round to slightly convoluted nuclei with open nuclear chromatin, a single to multiple prominent nucleoli, and variable amounts of basophilic cytoplasm (**Figure 2**). Immunohistochemistry demonstrated strong expression of the B-cell marker CD20 with coexpression of CD10 (weak), BCL6 and MUM1, corresponding to a germinal center B-cell (GCB) phenotype according to the Hans algorithm (11). The tumor also expressed BCL2 as well as MYC (heterogeneously). The lymphoblast marker TdT and the T-cell marker CD3 were negative. MIB1 staining showed a proliferation rate of more than 90%. Fluorescence *in situ* hybridization (FISH) of the BCL6, BCL2, MYC and IRF4/DUSP22 loci revealed a rearrangement of BCL6.

The final diagnosis was diffuse large B-cell lymphoma, not otherwise specified (DLBCL, NOS) of GCB subtype. Moreover, based on the localization and the lack of other lymphoma manifestations as shown in **Figure 1C**, the diagnosis corresponding to a primary extranodal manifestation of a DLBCL was made (10).

Further diagnostic procedures included CSF cytology, a second whole-body CT scan and a cerebral MRI scan. As shown in **Figure 1D**, the second CT scan 17 days after surgery revealed new nodular lymphoma manifestations in the inguinal region as well as in the right-sided frontal femoral compartment in line with a progression to systemic disease(see orange signs in **Figure 1D**). Nevertheless, enlarged lymph node or splenomegaly were not observed. CSF cytology revealed no malignant cells. The case was presented in our interdisciplinary lymphoma board. Taking into consideration the extensive systemic involvement and the unfavorable chance of a good recovery, only local palliative tumor control by radiotherapy was recommended. The patient subsequently received radiotherapy totaling 30 Gy in 10 fractions in the region of the upper thigh and groin including the external iliac lymph nodes. Radiotherapy was well tolerated and the patient could be discharged home with the support of a 24h nursing care service. Seven weeks later, a deep vein thrombosis in the right leg was diagnosed and was treated – in accordance with the national guidelines – with compression therapy and oral anticoagulation with apixaban without any further complications.

## 3 Interpretation

Neoplastic lesions affecting peripheral nerves are most often benign and, in experienced surgical hands, well-resectable (4, 12, 13). However, a minority of nerve sheath tumors reveal high-grade malignancies such as MPNST or rare entities that can diagnostically be difficult to distinguish based on imaging alone.

In the reported case, the geriatric patient presented with an acute monoplegia of the right lower extremity. MRI of the thigh and pelvis revealed an ill-defined contrast-enhancing lesion with central hemorrhage and perifocal edema. Due to the fast onset of symptoms, the radiological features, the initial inconspicuous CT scan and the lack of hematological abnormalities, an MPNST was firmly suspected. In consideration of the patient’s advanced age, the clinical presentation, and limited therapeutic options, metabolic imaging, such as FDG-PET as recommended by the ESMO guidelines for lymphoma staging (14), was not performed. We acknowledge, that this may have provided additional information on the dignity of the lesion and treatment response or might have helped to identify small systemic manifestations not visible on standard CT/MRI scan (**Figure 1C, D**) (9, 15). However, FDG uptake kinetics between MPNSTs and aggressive DLBCLs can hardly be distinguish (15, 16).

As shown in a retrospective study of benign peripheral nerve schwannomas of neurofibromatosis type 2 patients treated in our center, microsurgical resection has low complication rates, good functional preservation and offers good pain management (17). However, in a suspected malignant tumor, radical removal by en bloc resection according to surgical sarcoma criteria is mandatory and given the intraoperative appearance of a nerve destructing tumor compatible with a MPNST, en bloc resection was performed (9).

To our surprise, histopathology revealed an infiltrative destruction of the sciatic nerve by a DLBCL of GCB subtype, corresponding to the rare phenomenon historically known as neurolymphomatosis, i.e. a direct nerve infiltration by lymphoma cells (10, 18). Nerve infiltration can occur as the primary manifestations – like in the presented case – or secondary to other manifestations, with both occurring at approximately equal frequency in a mono-centric study (10). In line with this case, the most common lymphoma responsible is DLBCL (10, 19). Of note, although smaller studies and case reports have also described nerve infiltration by DLBCL of GCB subtype, recent large-scale analyses have revealed that extranodal manifestations like in the presented case more commonly occur in DLBCL with an activated B-cell-like gene expression signature (19–21).

The clinical presentation of the reported case is also in accordance with a published series of patients with nerve infiltration by lymphoma (19). A comprehensive diagnostic work-up including MRI and metabolic imaging as well as CSF cytology is recommended (19, 22). Due to its rarity, there are no established treatment algorithms and conclusive prospective clinical trials evaluating treatment options are missing. Therefore, therapeutic approaches are based on data available for DLBCL or primary central nervous system lymphoma (PCNSL) and individual treatment recommendations should be made based on a discussion in an interdisciplinary tumor board attended by neurooncologists as well as hematooncologists. Retrospective data reveal a tendency towards better long-term survival after including the anti-CD20 antibody Rituximab into treatment regimens (19). In the presented case, treatment consisted only of local tumor control by radiotherapy, taking the patient’s multimorbidity and frailty into consideration. Radiotherapy can offer effective local control in patients with relapsed DLBCL or with reduced life expectancy (23, 24).

## 4 Conclusion

Rarely, peripheral nerves can be directly infiltrated by hematologic neoplasms, which can present with similar clinical and radiological findings as the more common PNSTs. Comprehensive diagnostic algorithms including MRI as well as metabolic imaging like FDG-PET are recommended to evaluate the malignant potential of peripheral nerve lesions and rule out systemic or metastatic disease. A preoperative biopsy and histopathological assessment, if feasible, might be useful in lesions difficult to classify by imaging alone and might aid with surgical planning. Individual treatment recommendations should be made by an interdisciplinary tumor board, including both neurooncologists and, in case of a hematologic neoplasm, hematooncologists.

## 5 Patient perspective

At home the patient was able to mobilize herself independently with the orthosis. She was able to cover short distances of around 500 meters and, with the help of the nursing care service, was able to maintain her normal daily routines. She remained pain-compensated with the use of low-potency analgesics.

## Data availability statement

The original contributions presented in the study are included in the article/supplementary material. Further inquiries can be directed to the corresponding author.

## Ethics statement

Written informed consent was obtained from the individual(s) for the publication of any potentially identifiable images or data included in this article.

## Author contributions

HB: Conceptualization, Data curation, Formal analysis, Investigation, Visualization, Writing – original draft, Writing – review & editing. AV: Data curation, Formal analysis, Investigation, Visualization, Writing – original draft, Writing – review & editing. DF: Data curation, Formal analysis, Investigation, Writing – review & editing. AE: Data curation, Formal analysis, Investigation, Writing – review & editing. DT: Data curation, Formal analysis, Investigation, Writing – review & editing. JS: Data curation, Formal analysis, Investigation, Writing – review & editing. JM: Writing – review & editing. SK: Data curation, Formal analysis, Investigation, Writing – review & editing. FF: Conceptualization, Data curation, Formal analysis, Writing – review & editing. MT: Data curation, Formal analysis, Investigation, Writing – review & editing. MS: Data curation, Formal analysis, Investigation, Writing – review & editing. HH: Data curation, Formal analysis, Investigation, Writing – review & editing.
